# Association Between Hospital Private Equity Acquisition and Outcomes of Acute Medical Conditions Among Medicare Beneficiaries

**DOI:** 10.1001/jamanetworkopen.2022.9581

**Published:** 2022-04-29

**Authors:** Marcelo Cerullo, Kelly Yang, Karen E. Joynt Maddox, Ryan C. McDevitt, James W. Roberts, Anaeze C. Offodile

**Affiliations:** 1 Department of Surgery, Duke University, Durham, North Carolina; 2 National Clinician Scholars Program, jointly administered through Duke University and Durham Veterans Affairs Medical Center, Durham, North Carolina; 3 Department of Economics, Duke University, Durham, North Carolina; 4 Center for Health Economics and Policy, Institute for Public Health, Washington University in St Louis, St Louis, Missouri; 5 Division of Cardiology, Washington University School of Medicine, St Louis, Missouri; 6 Fuqua School of Business, Duke University, Durham, North Carolina; 7 National Bureau of Economic Research, Cambridge, Massachusetts; 8 Department of Plastic and Reconstructive Surgery, MD Anderson Cancer Center, Houston, Texas; 9 Baker Institute for Public Policy, Rice University, Houston, Texas; 10 Department of Health Services Research, MD Anderson Cancer Center, Houston, Texas

## Abstract

**Importance:**

As private equity (PE) acquisitions of short-term acute care hospitals (ACHs) continue, their impact on the care of medically vulnerable older adults remains largely unexplored.

**Objective:**

To investigate the association between PE acquisition of ACHs and access to care, patient outcomes, and spending among Medicare beneficiaries hospitalized with acute medical conditions.

**Design, Setting, and Participants:**

This cross-sectional study used a generalized difference-in-differences approach to compare 21 091 222 patients admitted to PE-acquired vs non–PE-acquired short-term ACHs between January 1, 2001, and December 31, 2018, at least 3 years before to 3 years after PE acquisition. The analysis was conducted between December 28, 2020, and February 1, 2022. Differences were estimated using both facility and hospital service area fixed effects. To assess the robustness of findings, regressions were reestimated after including fixed effects of patient county of origin to account for geographic differences in underlying health risks. Two subset analyses were also conducted: (1) an analysis including only hospitals in hospital referral regions with at least 1 PE acquisition and (2) an analysis stratified by participation in the Hospital Corporation of America 2006 acquisition. The study included Medicare beneficiaries 66 years and older who were hospitalized with 1 of 5 acute medical conditions: acute myocardial infarction (AMI), acute stroke, chronic obstructive pulmonary disease exacerbation, congestive heart failure exacerbation, and pneumonia.

**Exposures:**

Acquisition of hospitals by PE firms.

**Main Outcomes and Measures:**

Comorbidity burden (measured by Elixhauser comorbidity score), hospital length of stay, in-hospital mortality, 30-day mortality, 30-day readmission, and 30-day episode payments.

**Results:**

Among 21 091 222 total Medicare beneficiaries admitted to ACHs between 2001 and 2018, 20 431 486 patients received care at non–PE-acquired hospitals, and 659 736 received care at PE-acquired hospitals. Across all admissions, the mean (SD) age was 79.45 (7.95) years; 11 727 439 patients (55.6%) were male, and 4 550 012 patients (21.6%) had dual insurance; 2 996 560 (14.2%) patients were members of racial or ethnic minority groups, including 2 085 128 [9.9%] Black and 371 648 [1.8%] Hispanic; 18 094 662 patients (85.8%) were White. Overall, 3 083 760 patients (14.6%) were hospitalized with AMI, 2 835 777 (13.4%) with acute stroke, 3 674 477 (17.4%) with chronic obstructive pulmonary disease exacerbation, 5 868 034 (27.8%) with congestive heart failure exacerbation, and 5 629 174 (26.7%) with pneumonia. Comorbidity burden decreased slightly among patients admitted with acute stroke (difference, −0.04 SDs; 95% CI, −0.004 to −0.07 SDs) at acquired hospitals compared with nonacquired hospitals but was unchanged across the other 4 conditions. Among patients with AMI, a greater decrease in in-hospital mortality was observed in PE-acquired hospitals compared with non–PE-acquired hospitals (difference, −1.14 percentage points, 95% CI, −1.86 to −0.42 percentage points). In addition, a greater decrease in 30-day mortality (difference, −1.41 percentage points; 95% CI, −2.26 to −0.56 percentage points) was found at acquired vs nonacquired hospitals. However, 30-day spending and readmission rates remained unchanged across all conditions. The extent and directionality of estimates were preserved across all robustness assessments and subset analyses.

**Conclusions and Relevance:**

In this cross-sectional study using a difference-in-differences approach, PE acquisition had no substantial association with the patient-level outcomes examined, although it was associated with a moderate improvement in mortality among Medicare beneficiaries hospitalized with AMI.

## Key Points

**Question:**

What is the association between private equity (PE) acquisition of short-term acute care hospitals and measures of comorbidity, mortality, readmission, length of stay, and spending among Medicare beneficiaries admitted to the hospital with 1 of 5 acute medical conditions?

**Findings:**

In this cross-sectional study of more than 21 million Medicare beneficiaries with 5 different acute medical conditions who were hospitalized at short-term acute care hospitals, PE acquisition was associated with significantly lower inpatient mortality (−1.1 percentage points) and lower 30-day mortality (−1.4 percentage points) among patients admitted with acute myocardial infarction. However, PE acquisition was not associated with significant differences in other dimensions of quality and spending or with differences across other medical conditions.

**Meaning:**

The study’s findings suggest that PE acquisition has mixed consequences for patient-level outcomes overall but is associated with moderate and consistent improvement in mortality among Medicare beneficiaries hospitalized with acute myocardial infarction.

## Teaser

This cross-sectional study examines the association between private equity acquisition of short-term acute care hospitals and access to care, patient outcomes, and spending among Medicare beneficiaries hospitalized with acute medical conditions.

## Introduction

Private equity (PE) participation in the health care sector has increased over the past 20 years,^1,2,3^ with approximately one-half of these transactions occurring in the care delivery sector (physicians, hospitals, and nursing homes).^4^ Acute care hospitals (ACHs) are particularly attractive to PE firms; approximately 11% of all nongovernmental hospital discharges in 2017 were from a facility with a history of PE ownership.^5^ Private equity firms’ sustained interest in hospitals likely reflects several factors: (1) perceived inefficiencies that provide opportunities to improve operations,^2,6^ (2) an aging population that will require more acute care services,^7,8^ and (3) fragmented hospital markets that make horizontal consolidation a possible way to increase negotiating power over payers.^9,10,11^ These for-profit incentives have raised concerns about their consequences for the provision of health care services and the patient-practitioner relationship. The American College of Physicians recently disseminated a position paper calling for greater regulatory transparency and “longitudinal research on the effect of private equity investment on physicians’ clinical decision making, health care prices, access, and patient care, including the characteristics of models that may have adverse or positive effects on the quality and cost of care and the patient-physician relationship.”^12^

Recent work by Gupta et al^13^ estimated that PE ownership of nursing homes increased short-term mortality among Medicare patients by 10%, with concomitant reductions in other measures of patient well-being. In contrast, Braun et al^14^ estimated that, despite no consistent impact for spending or procedural volume, prices paid to dermatology practices increased by 3% to 5% after PE acquisition. However, short-term ACHs differ markedly from both of these subsectors. Despite substantial interest from policy makers noted in the Medicare Payment Advisory Commission’s June 2021 report,^15^ few studies have analyzed the association of PE acquisitions of ACHs with spending and clinical outcomes. Bruch et al^11^ identified modest but statistically significant improvements in risk-adjusted hospital-level quality measures for acute myocardial infarction (AMI) and pneumonia using data collected through the Centers for Medicare & Medicaid Services Hospital Compare program. The inconclusive findings of studies that have used aggregate quality measures and cost-to-charge ratios suggest the need for patient-level investigations to understand whether patient selection and/or differences in clinical practice bias are associated with changes in outcomes.

To address this knowledge gap, we examined the association between PE acquisition of short-term ACHs and outcomes among Medicare beneficiaries over an 18-year period using a difference-in-differences framework. We specifically sought to quantify the association of PE acquisitions using 6 important measures that encompass overall patient case mix and hospital clinical performance: comorbidity burden, inpatient mortality, 30-day mortality, 30-day readmission, inpatient length of stay (LOS), and 30-day episode spending.^16^ We examined this association across 5 common medical conditions: AMI, acute stroke, chronic obstructive pulmonary disease (COPD), congestive heart failure (CHF), and pneumonia. These 5 conditions account for a substantial portion of nonelective admissions, both broadly and among Medicare beneficiaries in particular.^17,18^

## Methods

### Study Population

We used the 100% Centers for Medicare & Medicaid Services standard analytic files and enrollment database to identify Medicare fee-for-service beneficiaries hospitalized between January 1, 2001, and December 31, 2018. The analysis was conducted between December 28, 2020, and February 1, 2022. This study was approved by the institutional review board of Duke University Medical Center. Informed consent was waived due to the deidentified nature of the data. This study followed the Strengthening the Reporting of Observational Studies in Epidemiology (STROBE) reporting guideline for cross-sectional studies.^19^

We identified patients 66 years and older who were admitted via the emergency department with a principal diagnosis of 1 of the following 5 conditions: AMI, acute stroke, CHF exacerbation, COPD exacerbation, and pneumonia. Medical conditions were identified using diagnostic codes from the *International Classification of Diseases, Ninth Revision*, and the *International Statistical Classification of Diseases and Related Health Problems, Tenth Revision *(eTable 1 in the Supplement). These cohorts were considered independently, and previously published protocols to classify index admissions and readmissions were used.^20^ Admissions classified as elective were excluded. To ensure that patients had at least 1 year of Medicare enrollment before hospital admission, those 65 years and younger were excluded.

Demographic information, including age, sex, race and ethnicity, and entitlement type, were abstracted from the beneficiary summary file. Comorbidities were identified using all admissions in the year before and up to the index admission and were summarized using Elixhauser comorbidity scores^21,22^; this approach was used because Centers for Medicare & Medicaid Services hierarchical conditions category risk scores were first implemented in 2004,^23^ and our period of interest included the years before its implementation.^24^

### Definition of Outcomes

Primary outcomes evaluated at the patient level included comorbidity burden (measured by Elixhauser comorbidity scores), in-hospital mortality, 30-day mortality (death within 30 days of admission), hospital LOS, 30-day all-cause readmission, and total inpatient spending per 30-day care episode. Hospital LOS and 30-day all-cause readmission were conditional on being discharged alive.

### Hospital Characteristics

Hospitals acquired by PE firms via primary or add-on leveraged buyout between 2003 and 2015 were identified using previously described methods.^5^ These hospitals were linked to financial data reported on Healthcare Cost Report Information System–Medicare Cost Reports and geographic location information (eg, physical address) contained in the Medicare provider of services files to determine patient county of origin, hospital service area (HSA), and hospital referral region. All non–federally owned hospitals with noncritical access that were not acquired by PE firms during this period were considered potential controls. Critical access hospitals were excluded because of their small inpatient bed count (<25 beds) and exemption from traditional reimbursement (ie, cost reimbursement rather than a prospective payment system) and fee structures. Hospital-level factors included size (<100 beds, 100-299 beds, or ≥300 beds), ownership type (for-profit, nonprofit, or government-run), teaching status (teaching vs nonteaching), medical school affiliation (affiliated vs unaffiliated), and core-based statistical area designation (metropolitan, micropolitan, or outside of a core-based statistical area designation).

### Statistical Analysis

The association of PE acquisition with patient outcomes was estimated using a generalized difference-in-differences approach covering a minimum of 3 years in the preacquisition (baseline) period and a 3-year limitation in the postacquisition period, using the interaction term between an indicator for PE acquisition and the 3-year period after acquisition. Our postacquisition horizon of 3 years was chosen to match the exit strategies (eg, divestment or secondary buyouts by another PE firm) commonly used by PE firms, which are not readily disclosed or identifiable in the public domain.

The following patient-level covariates were included in our model: age, sex, race and ethnicity (Asian or Pacific Islander, Black, Hispanic, North American Native, White, or other or unknown race and/or ethnicity), dual eligibility, type of entitlement (age, disability, or end-stage kidney disease), admission type (emergency or urgent), hospitalization within the previous year, and Elixhauser comorbidity score. Hospital fixed effects were included to account for time-invariant hospital-specific unobserved confounders; fixed effects for HSA by year were included to control for region-specific time trends, which encompassed either changes in overall hospital quality, legislation, or treatment standards introduced by clinical guidelines. Binary outcomes were estimated using a linear probability model, and continuous outcomes (LOS and total payments) were log-transformed and right winsorized at the 99th percentile to mitigate skewness (ie, values >99th percentile were set to the value of the 99th percentile).

In this specification, the difference-in-differences estimator can be interpreted as the difference in outcomes among patients within hospitals after PE acquisition, after adjustment for patient-level factors. Standard errors were clustered at the hospital level. We controlled for false discovery rate in the primary analyses using the Benjamini and Hochberg method, and we reported corrected *P* values alongside uncorrected *P* values.^25^ The full model specification is provided in eMethods in the Supplement, and results of the preparatory analyses using an event study framework to examine parallel trends are available in eFigures 1 to 6 in the Supplement. All analyses were conducted using Stata SE software, version 16.0 (StataCorp LLC), and a 2-tailed *P* < .05 was considered statistically significant.

Although our model specification allowed for a conservative estimate of the consequences of PE acquisition at the patient level, it may not have fully accounted for patient selection based on unobservable factors. In other words, differences detected within hospitals after PE acquisition may, in fact, have been associated with unobserved differences among patients admitted to those hospitals after acquisition. Therefore, we also considered an alternative specification that included fixed effects for a patient’s county of origin to account for geographic variation in health care access, social factors associated with health, and demographic factors that might have been associated with any noted differences in outcomes.^26,27,28^

We further examined 2 alternative specifications of the study cohort to assess whether the directionality of our estimates was consistent. First, because PE acquisitions are concentrated in specific geographic regions (eg, southeastern US) based on published literature,^5^ we repeated our analyses after restricting the sample to hospital referral regions in which at least 1 PE acquisition had occurred. Second, we stratified the sample of PE-acquired hospitals into 2 groups: members of the Hospital Corporation of America (HCA) health care system and all other hospitals. This decision was motivated by the fact that the 2006 HCA leveraged buyout by PE firms accounted for more than 50% of the hospitals in the sample, and stratification by HCA status was consistent with the approach used in recent PE scholarship.^11^ We repeated each generalized difference-in-differences model and excluded, in turn, HCA hospitals and non-HCA hospitals from the treatment group.

## Results

After accounting for observations in all years of our study period (2001-2018), a total of 21 091 222 care episodes were included across 3559 hospitals (257 of which were acquired by PE firms and had at least 3 years of data before and after acquisition; 11 hospitals closed within 3 years after acquisition). Overall, 20 431 486 episodes occurred at non–PE-acquired hospitals, and 659 736 occurred at PE-acquired hospitals. Across all admissions, the mean (SD) age was 79.45 (7.95) years; 11 727 439 patients (55.6%) were male, 9 363 783 (44.4%) were female, and 4 550 012 (21.6%) had dual insurance. Among the total of 21 091 222 patients, 2 996 560 (14.2%) were members of racial and ethnic minority groups (246 014 [1.2%] were Asian or Pacific Islander, 2 085 128 [9.9%] were Black, 371 648 [1.8%] were Hispanic, 73 348 [0.3%] were North American Native, and 220 422 [1.0%] were of unknown race and/or ethnicity), and 18 094 662 patients (85.8%) were White. A total of 3 083 760 patients (14.6%) were admitted with AMI, 2 835 777 (13.4%) with acute stroke, 5 868 034 (27.8%) with CHF exacerbation, 3 674 477 (17.4%) with COPD exacerbation, and 5 629 174 (26.7%) with pneumonia. Patient-level summary statistics across hospitals acquired and never acquired by PE firms are provided in the Table. Patient-level summary statistics across each of the 5 conditions (with data on age, sex, race and ethnicity, and Elixhauser comorbidity scores) at the beginning and end of our study period and annual rates for each outcome studied (in-hospital mortality, 30-day mortality, 30-day readmission, LOS, and spending) are provided in eTable 2 in the Supplement. Of note, unadjusted LOS and in-hospital mortality decreased across the study period for all 5 conditions. The proportion of patients receiving treatment across hospital types, including teaching vs nonteaching, for-profit vs nonprofit, and metropolitan vs micropolitan status are available in eTable 3 in the Supplement.

**Table.** Characteristics of Patients Treated in Hospitals Acquired and Not Acquired by PE Firms

Characteristic	No. (%)	
	Non–PE-acquired hospitals	PE-acquired hospitals
Total patients, No.	20 431 486	659 736
Sex		
Female	9 074 055 (44.4)	289 728 (43.9)
Male	11 357 431 (55.6)	370 008 (56.1)
Age, mean (SD), y	80.0 (8.0)	79.0 (8.0)
Race and ethnicity^a^		
Racial and ethnic minority groups^b^	2 888 883 (14.2)	107 677 (16.3)
White	17 542 603 (85.8)	552 059 (83.7)
Elixhauser comorbidity score, mean (SD)	2.08 (2.95)	2.16 (3.00)
Condition		
AMI	2 990 957 (14.6)	92 803 (14.1)
Acute stroke	2 756 284 (13.5)	79 493 (12.0)
CHF exacerbation	5 674 250 (27.8)	193 784 (29.4)
COPD exacerbation	3 556 147 (17.4)	118 330 (17.9)
Pneumonia	5 453 848 (26.7)	175 326 (26.6)
Hospital size		
<100 beds	2 072 976 (10.1)	41 784 (6.3)
100-299 beds	7 827 556 (38.3)	342 686 (51.9)
≥300 beds	10 530 954 (51.5)	275 266 (41.7)
Hospital teaching status		
Teaching	9 739 995 (47.7)	222 070 (33.7)
Nonteaching	10 691 491 (52.3)	437 666 (66.3)
Hospital ownership		
For-profit	1 677 888 (8.2)	463 867 (70.3)
Nonprofit	15 955 329 (78.1)	157 882 (23.9)
Government-run	2 798 269 (13.7)	37 987 (5.8)
Core-based statistical area designation		
Metropolitan	17 358 393 (85.0)	596 186 (90.4)
Micropolitan	2 360 853 (11.6)	49 847 (7.6)
Outside of core-based statistical area designation	712 240 (3.5)	13 703 (2.1)

Abbreviations: AMI, acute myocardial infarction; CHF, congestive heart failure; COPD, chronic obstructive pulmonary disease; PE, private equity.

^a^ Percentages for non–PE-acquired hospitals were calculated based on 20 431 486 total patients.

^b^ Racial and ethnic minority group category includes 246 014 patients who identified as Asian or Pacific Islander, 2 085 128 who identified as Black, 371 648 who identified as Hispanic, 73 348 who identified as North American Native, and 220 422 who were of unknown race and/or ethnicity.

### Patient Selection and Clinical Outcomes After PE Acquisition

**Figure 1.**
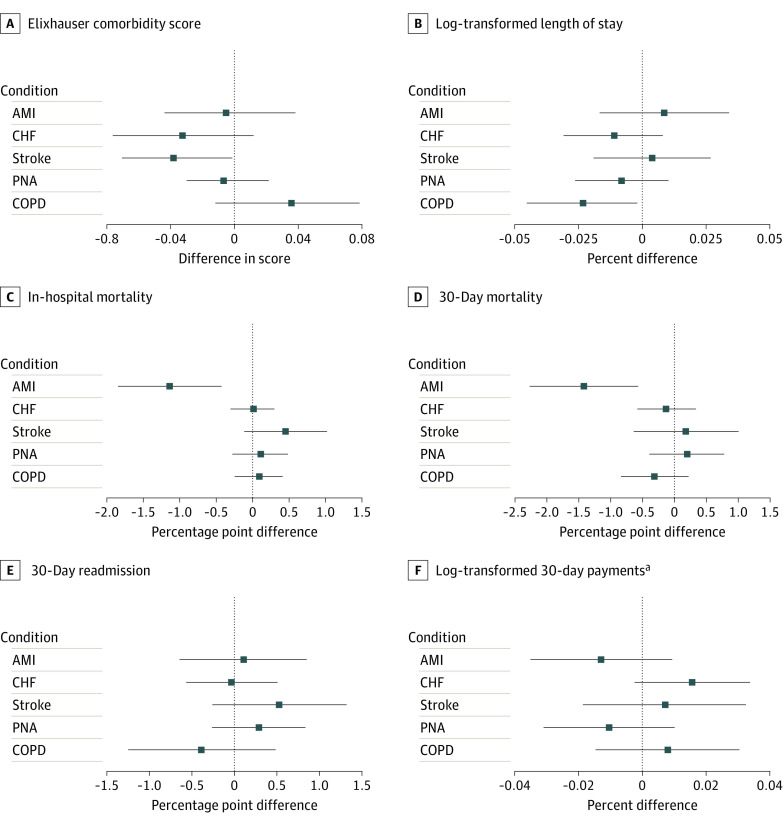
Difference-in-Differences Estimates for Primary Outcomes Across All 5 Medical Conditions Whiskers represent 95% CIs. AMI indicates acute myocardial infarction; CHF, congestive heart failure; COPD, chronic obstructive pulmonary disease; and PNA, pneumonia. ^a^Total inpatient payments per 30-day care episode.

The age at admission decreased slightly among patients hospitalized with pneumonia at PE-acquired hospitals compared with non–PE-acquired hospitals (difference, −0.20 SDs; 95% CI. −0.34 to −0.06 SDs; uncorrected *P* = .006; corrected *P* = .07) but was unchanged for the 4 other conditions examined. After PE acquisition, comorbidity burden decreased slightly among patients admitted with acute stroke (difference, −0.04 SDs; 95% CI, −0.004 to −0.07 SDs; uncorrected *P* = .03; corrected *P* = .24) at acquired hospitals compared with nonacquired hospitals but was unchanged across the other 4 conditions (Figure 1).

Among patients admitted with AMI, a greater decrease in in-hospital mortality was observed among PE-acquired hospitals compared with non–PE-acquired hospitals (difference, −1.14 percentage points; 95% CI, −1.86 to −0.42 percentage points; uncorrected *P* = .002; corrected *P* = .03). In addition, after PE acquisition, a −1.41 percentage point (95% CI, −2.26 to −0.56 percentage points; uncorrected *P* = .001; corrected *P* = .03) greater decrease in 30-day mortality was found at acquired hospitals compared with nonacquired hospitals. For the 4 other conditions examined, there were no differences in in-hospital mortality or 30-day mortality after PE acquisition. No differences in LOS were found among patients hospitalized with AMI, acute stroke, CHF, or pneumonia; patients admitted with COPD exacerbation had slightly shorter adjusted LOS after PE acquisition (difference, −2.34%; 95% CI, −4.52% to −0.15%; uncorrected *P* = .04; corrected *P* = .25), although this difference was not statistically significant after adjustment for false discovery rate. No differences in 30-day readmission and 30-day episode spending across all 5 conditions were noted (Figure 1).

### Robustness Assessments and Subset Analyses

**Figure 2.**
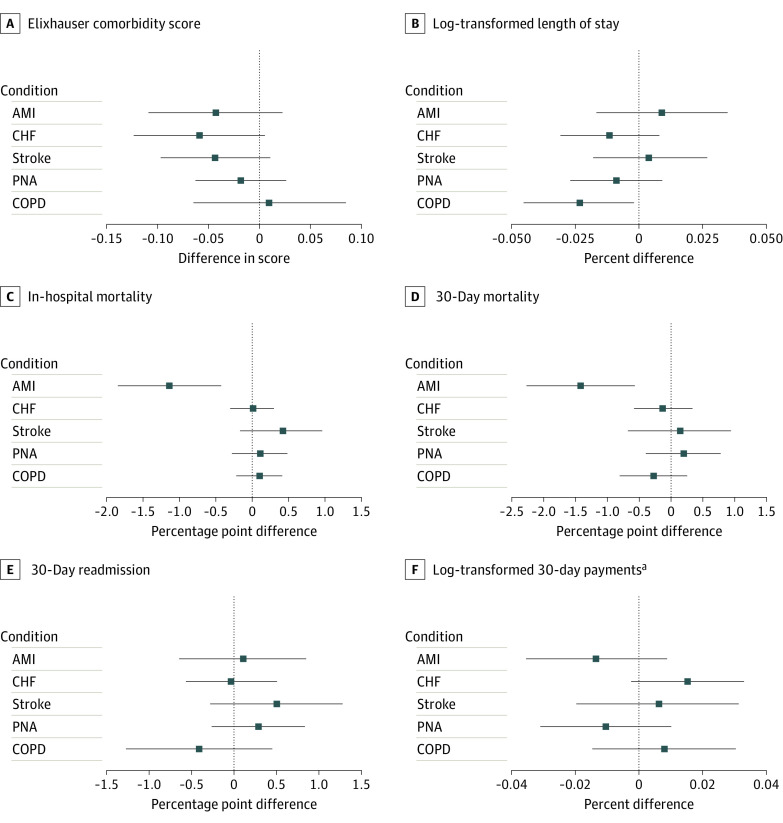
Difference-in-Differences Estimates After Including Fixed Effects of Patient County Whiskers represent 95% CIs. AMI indicates acute myocardial infarction; CHF, congestive heart failure; COPD, chronic obstructive pulmonary disease; and PNA, pneumonia. ^a^Total inpatient payments per 30-day care episode.

When fixed effects for beneficiary county of residence were included in addition to hospital fixed effects and hospital HSA-year fixed effects, difference-in-differences estimates of changes in in-hospital mortality (difference, −1.14 percentage points; 95% CI, −1.86 to −0.42 percentage points) and 30-day mortality (difference, −1.41 percentage points; 95% CI, −2.26 to −0.55 percentage points) among patients with AMI were consistent with the analysis using only hospital fixed effects and hospital HSA-year fixed effects (Figure 2). Moreover, when year fixed effects were included in lieu of hospital HSA-year fixed effects, difference-in-differences estimates among patients admitted with AMI were consistent with those obtained using HSA-year fixed effects; specifically, decreases of 0.74 percentage points (95% CI, −1.19 to −0.29 percentage points) in in-hospital mortality and 0.94 percentage points (95% CI, −1.51 to −0.37 percentage points) in 30-day mortality were observed. Hospital LOS among patients admitted with COPD exacerbation was similarly shorter (difference, −1.88%; 95% CI, −3.40% to −0.33%), although the clinical importance of this was not clear.

After restricting the patient cohort to those who received treatment at hospitals in hospital referral regions with at least 1 PE acquisition, patients admitted with AMI had a greater decrease in in-hospital mortality (difference, −1.12 percentage points; 95% CI, −1.89 to −0.45 percentage points) and 30-day mortality (difference, −1.41 percentage points; 95% CI, −2.26 to −0.57 percentage points) after PE acquisition. In addition, LOS among patients admitted with COPD exacerbation similarly decreased (difference, −2.43%; 95% CI, −4.57% to −0.30%), although the extent of the estimate was small (eFigure 7 in the Supplement).

After partitioning hospitals based on whether they were part of the 2006 HCA acquisition, the directionality of significant findings remained consistent. Among HCA hospitals, patients with AMI experienced a greater decrease in in-hospital mortality (difference, −1.33 percentage points; 95% CI, −2.19 to −0.47 percentage points) and 30-day mortality (difference, −1.40 percentage points; 95% CI, −2.39 to −0.40 percentage points). Estimates for these 2 outcomes in non-HCA hospitals were similar in extent but not statistically significant (AMI in-hospital mortality: −0.59 percentage points [95% CI, −1.96 to 0.78 percentage points]; 30-day mortality: −1.10 percentage points [95% CI, −2.74 to 0.54 percentage points]). Among non-HCA hospitals, LOS among patients admitted with COPD exacerbation was lower after acquisition (difference, −4.29%; 95% CI, −8.26% to −0.32%), as was LOS among patients admitted with CHF (difference, −4.51%; 95% CI, −7.72% to −1.29%) (eFigure 8 in the Supplement).

## Discussion

This cross-sectional study used a difference-in-differences framework to evaluate the quality of care at PE-owned ACHs relative to ACHs without a history of PE acquisition. The impact of PE acquisition for case selection and clinical quality at short-term ACHs remains relevant to ongoing policy discussions aimed at promoting greater value in health care spending. Among Medicare beneficiaries, PE acquisition was associated with consistent improvements in both in-hospital and 30-day mortality among patients with AMI, with comparable overall spending. We found a small but likely clinically nonmeaningful decrease in LOS among patients admitted with COPD exacerbation and no difference in 30-day spending or 30-day readmission for all 5 conditions studied. We did not find any evidence of systematic upcoding (ie, submission of diagnostic codes for services more expensive than those actually provided) or increased intensity in comorbidity coding (ie, more frequent comorbidity coding and/or submission of higher comorbidity scores) in the postacquisition period. These findings were largely corroborated by 2 separate robustness assessments, which included specifications accounting for any unobserved variation over time or between patients’ socioeconomic status. These findings also persisted in subset analyses that restricted the study cohort to patients who received treatment in hospital referral regions that had any PE activity, and the directionality of our results persisted when hospitals were stratified by their participation in the 2006 HCA leveraged buyout.

This study’s findings were inconsistent with the prevailing concerns surrounding PE acquisitions of health care systems, perhaps highlighting the need for nuanced investigations into the role of for-profit investments in health care. Regulators may determine that it is not sufficient that for-profit institutions do no harm; they may instead decide that for-profit owners produce improvements in value of care, either through better outcomes, lower costs, or both. Proponents of PE acquisition often assert that revenue generation from target hospitals via taxation is a societal boon, while the patients they serve may benefit from economies of scale, management expertise, and an incentive to implement cost-effective care.^5,7,29,30,31^ Critics assert that PE firms, unlike other for-profit institutions, have an inherent incentive to favor short-term returns rather than long-term investments (eg, information technologies and care redesign) that would otherwise meaningfully improve population health.^29,32^ In certain aspects of health care services, the latter view has been bolstered by important research^13,14,33,34^ in nursing homes and outpatient clinical practices. Private equity–owned nursing facilities performed comparably, both in terms of equipment shortages and resident outbreaks, during the COVID-19 pandemic.^14,33^ However, cost-cutting measures that led to unsafe staffing ratios resulted in worse patient outcomes,^13^ and increased market power has made outpatient practices more costly for payers.^34^ Concerns about similar impacts in hospitals are therefore justified. Rather than focus on PE acquisitions of hospitals as a distinct problem in the health care delivery sector, perhaps PE activity might be viewed as a proxy for market failures and payment loopholes that can be exploited (eg, surprise billing [unexpected charges from out-of-network hospitals or practitioners], horizontal consolidation, Medicare payment differentials for physician-administered drugs under Part B, and Medicare Advantage upcoding for benchmark payments [in which enrollees' potentially comorbid diagnoses are recorded to increase risk-adjusted payments]).^35^

Our findings with respect to the improvements in AMI care after PE acquisition were consistent with those of Bruch et al.^11^ Acute MI may represent an ideal clinical condition for targeted quality improvement and care redesign efforts by PE ownership because of (1) clear guidelines from the American College of Cardiology/American Heart Association on the proper management of patients with AMI,^36^ (2) a well-understood association between guideline adherence and improved outcomes,^37^ and (3) the resource-intensive nature of AMI identification and treatment (eg, diagnostic imaging, cardiac catheterization, and percutaneous coronary intervention).^38^

There are several possible explanations for our findings. First, the variation in management practices and capital allocation across PE firms may result in substantively different operational changes after acquisition and may therefore have variable consequences for outcomes. Another explanation may be that all short-term ACHs, PE-acquired or not, are subject to the same regulatory oversight, accreditation, and quality reporting environment.^39^ Although PE ownership has been associated with operational changes,^40^ it is possible that extant regulatory tolerance for adverse outcomes prevents drastic cost-cutting measures that result in worse clinical care. At the same time, there may not be clear avenues for quality improvement in the 4 other medical conditions examined in the present study. Quality measures for those conditions have several benchmarking approaches that are not as universally accepted or as easily identifiable in Medicare claims (eg, oxygen assessment, antimicrobial timing, and appropriate initial antimicrobial selection for pneumonia).^41,42^ Within the broader literature examining hospital for-profit conversions and their association with clinical outcomes, our results were also consistent with work by Joynt et al,^32^ which found no difference in 30-day risk-adjusted mortality rates and process quality factors associated with AMI, CHF, and pneumonia across 237 hospitals that converted to for-profit status between 2003 and 2010.

### Limitations

This study has several limitations. First, we consider all PE acquisitions to be the same type of exposure. Although PE firms structure their investments in different ways, we focused on leveraged buyouts in this study because they are the most common type, and this focus allowed us to avoid confounding in our analysis that could have occurred by including other deal structures. However, the amount of debt burden passed on to acquired hospitals by PE firms (ie, the financial obligation resulting from the deal that has implications for the extent of cost-cutting or revenue increases required to remain solvent) varies and is often not accessible in the public domain. Moreover, oversight by state regulators and overall investment practices of each firm may vary, which could result in nonuniform consequences for each acquisition.

Second, although we consider a broad set of medical conditions and quality measures, we are unable to capture all dimensions of care quality.^43^ Third, a difference-in-differences analysis in which the treatment exposure is a nonrandom event (ie, PE acquisition) may be subject to selection bias.^43^ Cross-sectional analyses of PE-owned hospitals have revealed that PE firms have lower staffing levels for a standard measure of patient burden.^44^ We posit that the nature of nonelective admissions renders our findings less subject to patient selection or practitioner-related discrimination; future studies can further assess these differences in the likelihood of acquisition to estimate the impact of PE acquisition for changes in elective admissions.^45^

Fourth, our study was conducted entirely among Medicare beneficiaries; although subtle shifts in the total proportion of Medicare patients may be observable, profit-seeking via changes in patient access (ie, guiding Medicare or uninsured patients away from inpatient care after PE acquisition or reducing visits or readmissions among high-risk patients with complex conditions) and the consequences of vertical integration (as with the Medicare Advantage program) cannot be easily measured. Fifth, we examined a short period (3 years) after PE acquisition to avoid overstating or misassigning the consequences of PE ownership by incorporating data after the inevitable sale (exit) by the PE firm, which usually occurs at 5 to 7 years after acquisition. Therefore, our findings cannot be generalized to the longer-term consequences of PE acquisition.^46^

## Conclusions

In this cross-sectional study, acquisition of short-term ACHs by PE firms was associated with modest improvements in measures of mortality for AMI, with no changes in mortality outcomes across 4 other medical conditions that account for a large proportion of nonelective admissions among Medicare beneficiaries. These findings challenge the narrative that PE investments in all subsectors of health care delivery organizations increase health spending and systematically worsen quality. We believe these findings may further motivate longitudinal research on the consequences of PE acquisition for physician decision-making, access to care, and health care prices.^12^ Preference-sensitive elective admissions with clinical management and profitability that are more dependent on patient selection represent a rich clinical context for this inquiry.

## Notes

Supplement. **eMethods.** Additional Description of Study Methods **eTable 1.** Primary Diagnosis Codes **eTable 2.** Patient-Level Summary Statistics Across Conditions at the Beginning and End of the Study Period **eTable 3.** Distribution of Patients by Treating Hospital Characteristics Across Conditions at the Beginning and End of the Study Period **eFigure 1.** Event Study Plot for Patient Comorbidity (Elixhauser) **eFigure 2.** Event Study Plot of Differences in Log-Transformed Length of Stay **eFigure 3.** Event Study Plot for Probability of In-Hospital Mortality **eFigure 4.** Event Study Plot for Probability of 30-Day Mortality **eFigure 5.** Event Study Plot for Probability of 30-Day Readmission **eFigure 6.** Event Study Plot for Log-Transformed 30-Day Episode Payments **eFigure 7.** Difference-in-Differences Estimates in Subsample of Hospital Referral Regions With at Least 1 Private Equity Acquisition **eFigure 8.** Difference-in-Differences Estimates for HCA and Non-HCA Hospitals
