# Supplementary material for: Association Between Hospital Private Equity Acquisition and Outcomes of Acute Medical Conditions Among Medicare Beneficiaries
Source: JAMA Netw Open. 2022 Apr 29;5(4):e229581. doi: 10.1001/jamanetworkopen.2022.9581 (PMC9055457; doi:10.1001/jamanetworkopen.2022.9581)
Supplement: Supplement. — eMethods. Additional Description of Study Methods eTable 1. Primary Diagnosis Codes eTable 2. Patient-Level Summary Statistics Across Conditions at the Beginning and End of the Study Period eTable 3. Distribution of Patients by Treating Hospital Characteristics Across Conditions at the Beginning and End of the Study Period eFigure 1. Event Study Plot for Patient Comorbidity (Elixhauser) eFigure 2. Event Study Plot of Differences in Log-Transformed Length of Stay eFigure 3. Event Study Plot for Probability of In-Hospital Mortality eFigure 4. Event Study Plot for Probability of 30-Day Mortality eFigure 5. Event Study Plot for Probability of 30-Day Readmission eFigure 6. Event Study Plot for Log-Transformed 30-Day Episode Payments eFigure 7. Difference-in-Differences Estimates in Subsample of Hospital Referral Regions With at Least 1 Private Equity Acquisition eFigure 8. Difference-in-Differences Estimates for HCA and Non-HCA Hospitals [file jamanetwopen-e229581-s001.pdf]

Supplementary Online Content

Cerullo M, Yang K, Joynt Maddox KE, McDevitt RC, Roberts JW, Offodile AC 2nd. Association between hospital private equity acquisition and outcomes of acute medical conditions among Medicare beneficiaries. *JAMA Netw Open*. 2022;5(4):e229581. doi:10.1001/jamanetworkopen.2022.9581

eMethods. Additional Description of Study Methods

eTable 1. Primary Diagnosis Codes

eTable 2. Patient-Level Summary Statistics Across Conditions at the Beginning and End of the Study Period

eTable 3. Distribution of Patients by Treating Hospital Characteristics Across Conditions at the Beginning and End of the Study Period

eFigure 1. Event Study Plot for Patient Comorbidity (Elixhauser)

eFigure 2. Event Study Plot of Differences in Log-Transformed Length of Stay

eFigure 3. Event Study Plot for Probability of In-Hospital Mortality

eFigure 4. Event Study Plot for Probability of 30-Day Mortality

eFigure 5. Event Study Plot for Probability of 30-Day Readmission

eFigure 6. Event Study Plot for Log-Transformed 30-Day Episode Payments

eFigure 7. Difference-in-Differences Estimates in Subsample of Hospital Referral Regions With at Least 1 Private Equity Acquisition

eFigure 8. Difference-in-Differences Estimates for HCA and Non-HCA Hospitals

This supplementary material has been provided by the authors to give readers additional information about their work.

© 2022 Cerullo M et al. *JAMA Network Open*.

eMethods. Additional Description of Study Methods

Event study framework

We estimate the period-specific effect of each year relative to acquisition for hospitals acquired by private equity firms the following specification:

$$Y_{ijt} = PE_j \sum_{\substack{s=-6 \\ s\neq -1}}^{6} \gamma_s 1\{t - t_j^* = s\} + X_{ijt}\beta + \mu_j + \delta_{m(j)t} + \epsilon_{ijt}$$

In this instance, Y_{ijt} is the outcome measure for a given patient i at hospital j in year t. The indicators $1\{t - t_j^* = s\}$ reflect the time relative to the acquisition year t_j^*, and are zeroes in all years for hospitals that are not acquired by a private equity firm. Data from years greater than five years relative to the year of acquisition (i.e., $|s| \geq 6$), are grouped into a single indicator. The parameter γ_s is the pre- or post-treatment period coefficient. The coefficients for the pre-treatment periods (here given by $\gamma_{-6}, \gamma_{-5}, \gamma_{-4}, \gamma_{-3}$, and γ_{-2}) can be visually examined for parallel trends and formally tested for equality. All years of data were kept in the event-study sample, and the year prior to PE acquisition (i.e., $s = -1$), is the omitted category.

The second term (including the parameter vector β), includes a vector of patient-level controls, X_{ijt}, which include patient age, Elixhauser comorbidity index, and indicators for race, sex, admission and entitlement type, and an indicator for any hospitalization in the past year. Hospital fixed effects (μ_j) and market (hospital service area [HSA]) by year fixed effects $\delta_{m(j)t}$, control for time-invariant hospital-specific unobservables and market-specific time trends, respectively. Standard errors are clustered at the hospital level.

Difference-in-differences analysis

We estimate the reduced-form effect of private equity acquisition on patient outcomes using the following difference-in-differences specification:

$$Y_{ijt} = \gamma(PE_j \times Post_t) + X_{ijt}\beta + \mu_j + \delta_{m(j)t} + \epsilon_{ijt}$$

As before, Y_{ijt} is the outcome measure for a given patient i at hospital j in year t. PE_j is an indicator for the exposure of interest (private equity acquisition) that takes a value of 1 for hospitals which are acquired by a private equity firm, and $Post_t$ is an indicator that takes a value of 1 in each year following the acquisition. The parameter γ is the difference-in-differences estimator, which quantifies the effect of private equity acquisition. As above in the event study framework, the second term in the estimating equation (which estimates the parameter vector β), includes a vector of patient-level controls, X_{ijt}. Hospital fixed effects (μ_j) and market (hospital service area [HSA]) by year fixed effects $\delta_{m(j)t}$, control for time-invariant hospital-specific unobservables and market-specific time trends, respectively. Standard errors are clustered at the hospital level.

All hospitals acquired between 2001 and 2018 are included in the potential treatment group. Observations within three years prior to and following the acquisition were included. Observations from the acquisition year were excluded. All observations from all years of the study period for all potential control hospitals were included. The full sample was restricted to hospitals defined as short-term acute care hospitals (American Hospital Association service code ("SERV" variable) 10), excluding hospitals with a critical access designation.

© 2022 Cerullo M et al. *JAMA Network Open*.

eTable 1. Primary Diagnosis Codes

	Format	Codes
**Acute myocardial infarction**	ICD-9-CM	410.00, 410.01, 410.10, 410.11, 410.20, 410.21, 410.30, 410.31, 410.40, 410.41, 410.50, 410.51, 410.60, 410.61, 410.70, 410.71, 410.80, 410.81, 410.90, 410.91
	ICD-10	I21.11, I21.19, I21.21, I21.29, I21.3, I21.4, I22.1, I22.2, I22.29, I22.8, I22.9
**Congestive Heart Failure**	ICD-9-CM	428.0, 428.1, 428.20, 428.21, 428.22, 428.23, 428.30, 428.31, 428.32, 428.33, 428.40, 428.41, 428.42, 428.43, 428.9
	ICD-10-CM	I50.1, I50.20, I50.21, I50.30, I50.31, I50.32, I50.33, I50.40, I50.41, I50.42, I50.43, I50.9
**Acute Stroke**	ICD-9-CM	433.01, 433.11, 433.21, 433.31, 433.81, 433.91, 434.01, 434.11, 434.91
	ICD-10-CM	I63.00, I63.011, I63.012, I63.013, I63.019, I63.02, I63.031, I63.032, I63.033, I63.039, I63.09, I63.10, I63.111, I63.112, I63.113, I63.119, I63.12, I63.131, I63.132, I63.133, I63.139, I63.19, I63.20, I63.211, I63.212, I63.213, I63.219, I63.22, I63.231, I63.232, I63.233, I63.239, I63.29, I63.30, I63.311, I63.312, I63.313, I63.319, I63.321, I63.322, I63.323, I63.329, I63.331, I63.332, I63.333, I63.339, I63.341, I63.342, I63.343, I63.349, I63.39, I63.40, I63.411, I63.412, I63.413, I63.419, I63.421, I63.422, I63.423, I63.429, I63.431, I63.432, I63.433, I63.439, I63.441, I63.442, I63.443, I63.449, I63.49, I63.50, I63.511, I63.512, I63513, I63.519, I63.521, I63.522, I63.523, I63.529, I63.531, I63.532, I63.533, I63.539, I63.541, I63.542, I63.543, I63.549, I63.59, I63.6, I63.81, I63.89, I63.9
**Pneumonia**	ICD-9-CM	481, 482.0, 482.1, 482.2, 482.30, 482.31, 482.32, 482.39, 482.40, 482.41, 482.42, 482.49, 482.82, 482.83, 482.84, 482.89, 482.9, 483.1, 483.8, 485, 486
	ICD-10-CM	A481, J14, J15.20, J15.211, J15.212, J15.29, J15.4, J15.5, J15.6, J15.7, J15.8, J15.9, J16.0, J16.8, J18.0, J18.8, J18.9
**Chronic obstructive pulmonary disease**	ICD-9-CM	490, 491.0, 491.1, 491.2, 491.20, 491.21, 491.22, 491.8, 491.9, 492.0, 492.8, 494, 494.0, 494.1, 496
	ICD-10-CM	J40, J41.0, J41.1, J41.8, J42, J43.0, J43.1, J43.2, J43.8 , J43.9, J44.0, J44.1, J44.9, J47.0, J47.1, J47.9

© 2022 Cerullo M et al. *JAMA Network Open*.

eTable 2. Patient-Level Summary Statistics Across Conditions at the Beginning and End of the Study Period

	AMI		CHF		Stroke		PNA		COPD	
	2001	2018	2001	2018	2001	2018	2001	2018	2001	2018
Elixhauser Comorbidity Index	1.352	1.702	2.301	2.831	1.254	1.606	1.276	2.042	2.158	2.967
	(2.233)	(2.994)	(2.895)	(3.656)	(2.112)	(2.845)	(2.214)	(3.176)	(2.528)	(3.572)
Length of stay, (days)	6.125	4.404	5.586	4.724	6.415	4.313	6.576	4.757	5.376	4.168
	(5.306)	(4.373)	(4.652)	(3.734)	(5.132)	(3.969)	(5.114)	(3.607)	(4.192)	(3.178)
In-hospital mortality	0.129	0.0585	0.0559	0.0358	0.0806	0.0426	0.0899	0.0312	0.0364	0.0129
	(0.335)	(0.235)	(0.230)	(0.186)	(0.272)	(0.202)	(0.286)	(0.174)	(0.187)	(0.113)
30-day mortality	0.190	0.118	0.114	0.132	0.157	0.136	0.157	0.107	0.0816	0.0534
	(0.392)	(0.323)	(0.318)	(0.338)	(0.364)	(0.343)	(0.364)	(0.309)	(0.274)	(0.225)
30-day readmission	0.192	0.161	0.174	0.179	0.148	0.131	0.112	0.142	0.192	0.178
	(0.394)	(0.367)	(0.379)	(0.384)	(0.355)	(0.337)	(0.315)	(0.349)	(0.394)	(0.383)
Medicare spending: 30-day	12069	27696	6306	25044	9469	23960	4961	24346	5823	15790
	(14430)	(25256)	(10348)	(25159)	(10938)	(21553)	(9798)	(23670)	(8075)	(15331)
Age at admission, (years)	78.95	77.85	79.93	81.25	79.35	79.62	80.41	80.11	76.70	76.86
	(7.643)	(8.278)	(7.654)	(8.669)	(7.438)	(8.486)	(7.874)	(8.512)	(6.864)	(7.502)
Proportion male	0.481	0.556	0.402	0.431	0.396	0.449	0.452	0.455	0.435	0.419
	(0.500)	(0.497)	(0.490)	(0.495)	(0.489)	(0.497)	(0.498)	(0.498)	(0.496)	(0.493)
Proportion white	0.891	0.848	0.843	0.896	0.841	0.819	0.879	0.865	0.892	0.858
	(0.312)	(0.359)	(0.364)	(0.305)	(0.366)	(0.385)	(0.326)	(0.342)	(0.310)	(0.349)
Proportion black	0.0757	0.0845	0.120	0.0630	0.122	0.117	0.0828	0.0782	0.0779	0.0981
	(0.265)	(0.278)	(0.325)	(0.243)	(0.328)	(0.322)	(0.276)	(0.268)	(0.268)	(0.297)
Proportion admitted in preceding year	0.341	0.282	0.472	0.436	0.346	0.290	0.325	0.352	0.539	0.491
	(0.474)	(0.450)	(0.499)	(0.496)	(0.476)	(0.454)	(0.468)	(0.477)	(0.498)	(0.500)

*Values are means and standard deviations.

© 2022 Cerullo M et al. *JAMA Network Open*.

eTable 3. Distribution of Patients by Treating Hospital Characteristics Across Conditions at the Beginning and End of the Study Period

	AMI		CHF		Stroke		PNA		COPD	
	2001	2018	2001	2018	2001	2018	2001	2018	2001	2018
Total beds*	364.6	417.3	362.2	352.3	382.3	456.4	332.3	368.2	334.1	365.4
	(260.4)	(319.3)	(267.1)	(305.2)	(270.3)	(345.9)	(255.6)	(319.4)	(250.9)	(309.5)
Proportion that are non-profit	0.772	0.729	0.761	0.740	0.775	0.743	0.748	0.719	0.735	0.715
	(0.420)	(0.445)	(0.426)	(0.439)	(0.417)	(0.437)	(0.434)	(0.449)	(0.441)	(0.451)
Proportion that are for profit	0.101	0.161	0.110	0.112	0.0979	0.132	0.113	0.153	0.121	0.158
	(0.301)	(0.367)	(0.313)	(0.315)	(0.297)	(0.339)	(0.316)	(0.360)	(0.326)	(0.365)
Proportion that are government-run	0.127	0.111	0.128	0.148	0.127	0.125	0.139	0.127	0.144	0.127
	(0.333)	(0.314)	(0.335)	(0.355)	(0.333)	(0.331)	(0.346)	(0.333)	(0.351)	(0.333)
Proportion that are major teaching hospitals	0.376	0.431	0.367	0.352	0.379	0.430	0.341	0.381	0.340	0.372
	(0.484)	(0.495)	(0.482)	(0.477)	(0.485)	(0.495)	(0.474)	(0.486)	(0.474)	(0.483)
Proportion that are minor teaching hospitals	0.0847	0.158	0.0925	0.150	0.102	0.193	0.0773	0.120	0.0699	0.136
	(0.278)	(0.365)	(0.290)	(0.357)	(0.303)	(0.395)	(0.267)	(0.325)	(0.255)	(0.343)
Proportion with a core-based statistical area designated “metropolitan”	0.844	0.904	0.843	0.823	0.868	0.919	0.807	0.846	0.808	0.844
	(0.362)	(0.295)	(0.363)	(0.382)	(0.338)	(0.273)	(0.395)	(0.361)	(0.394)	(0.363)
Proportion with a core-based statistical area designated “micropolitan”	0.123	0.0855	0.118	0.142	0.107	0.0703	0.144	0.124	0.142	0.123
	(0.328)	(0.280)	(0.323)	(0.349)	(0.309)	(0.256)	(0.351)	(0.329)	(0.349)	(0.329)

*Values are means and standard deviations.

© 2022 Cerullo M et al. *JAMA Network Open*.

eFigure 1. Event Study Plot for Patient Comorbidity (Elixhauser)

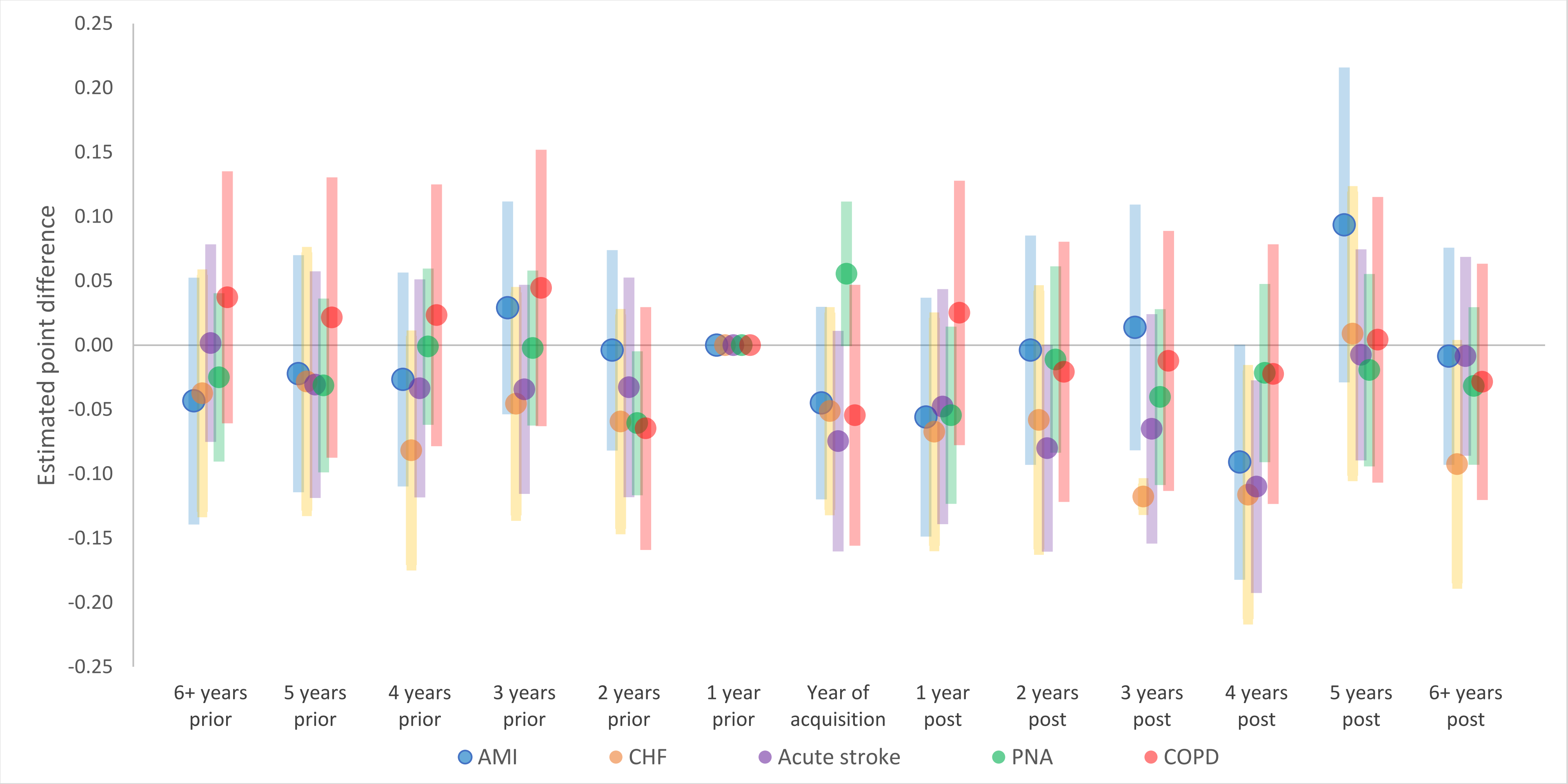

Condition	Pre-acquisition mean	Pre-trend p-value (5-year horizon)	Pre-trend p-value (4-year horizon)
AMI	1.588	0.72	0.73
CHF	2.897	0.48	0.34
Stroke	1.527	0.87	0.91
PNA	1.634	0.26	0.17
COPD	2.602	0.32	0.24

© 2022 Cerullo M et al. *JAMA Network Open*.

eFigure 2. Event Study Plot of Differences in Log-Transformed Length of Stay

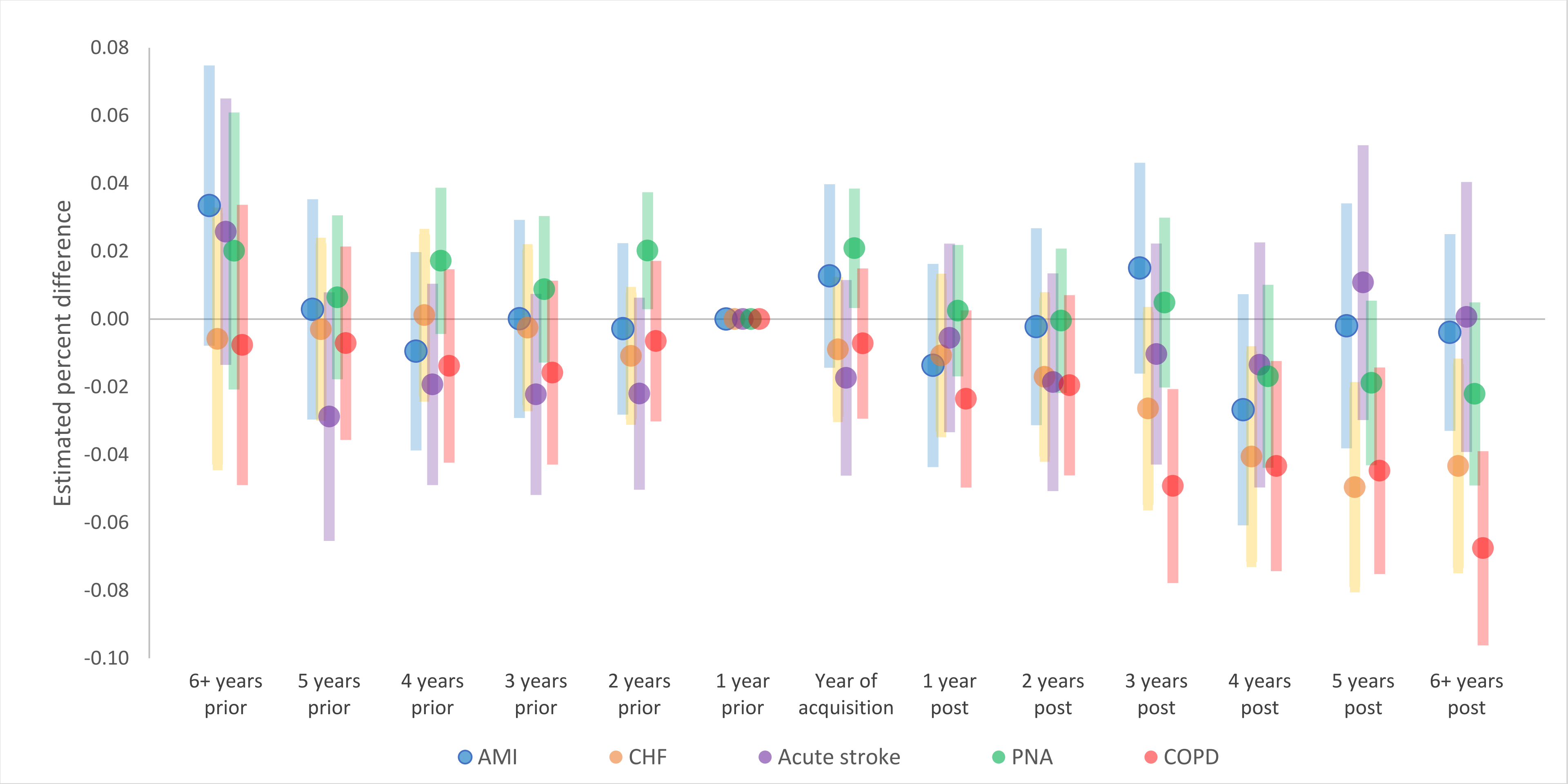

Condition	Pre-acquisition mean	Pre-trend p-value (5-year horizon)	Pre-trend p-value (4-year horizon)
AMI	1.449	0.49	0.90
CHF	1.458	0.75	0.62
Stroke	1.446	0.10	0.53
PNA	1.573	0.18	0.13
COPD	1.379	0.90	0.82

© 2022 Cerullo M et al. *JAMA Network Open*.

eFigure 3. Event Study Plot for Probability of In-Hospital Mortality

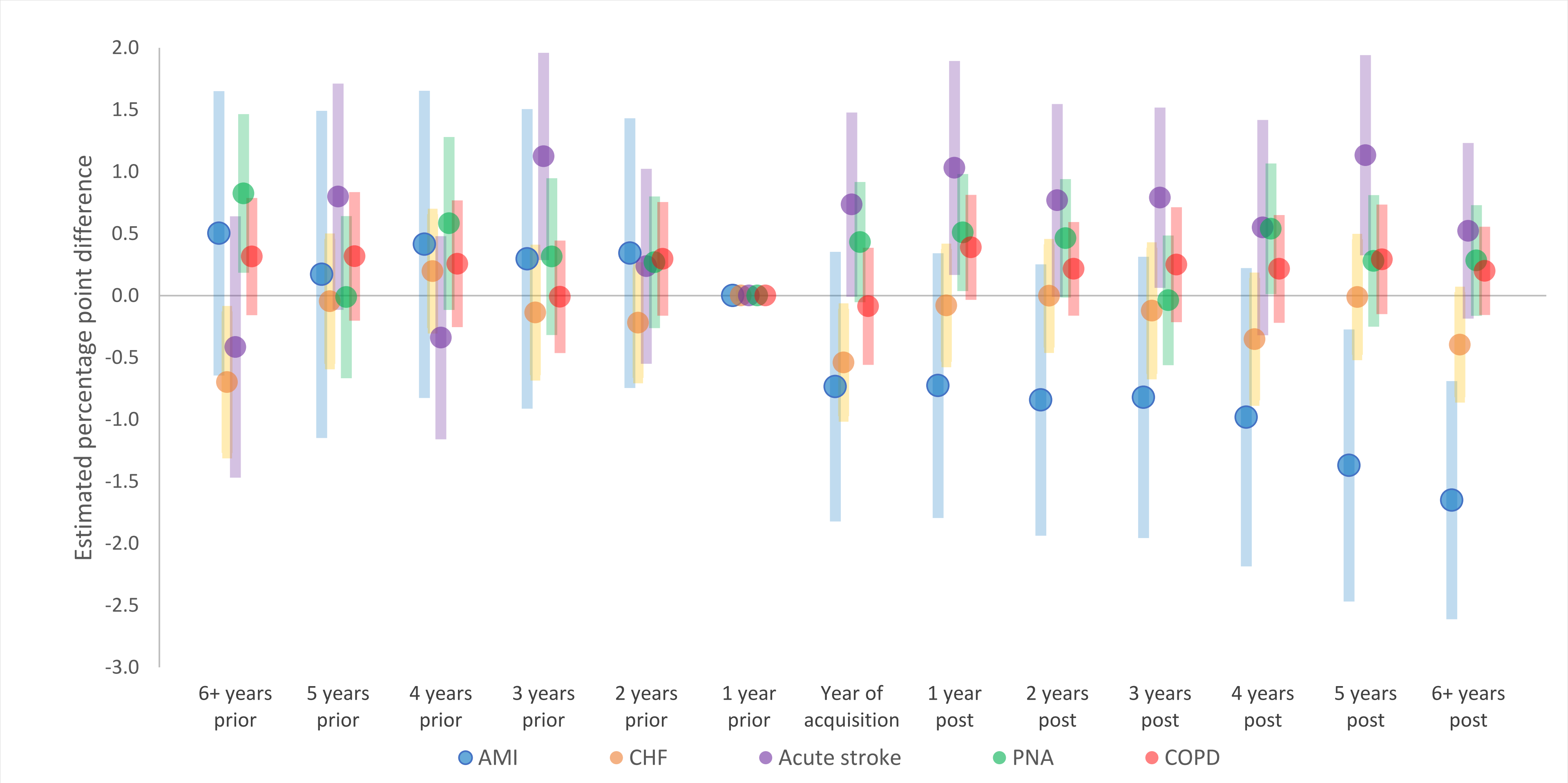

Condition	Pre-acquisition mean	Pre-trend p-value (5-year horizon)	Pre-trend p-value (4-year horizon)
AMI	10.4	0.96	0.96
CHF	4.2	0.04	0.57
Stroke	5.2	<0.01	<0.01
PNA	5.1	0.12	0.41
COPD	1.9	0.56	0.50

© 2022 Cerullo M et al. *JAMA Network Open*.

eFigure 4. Event Study Plot for Probability of 30-Day Mortality

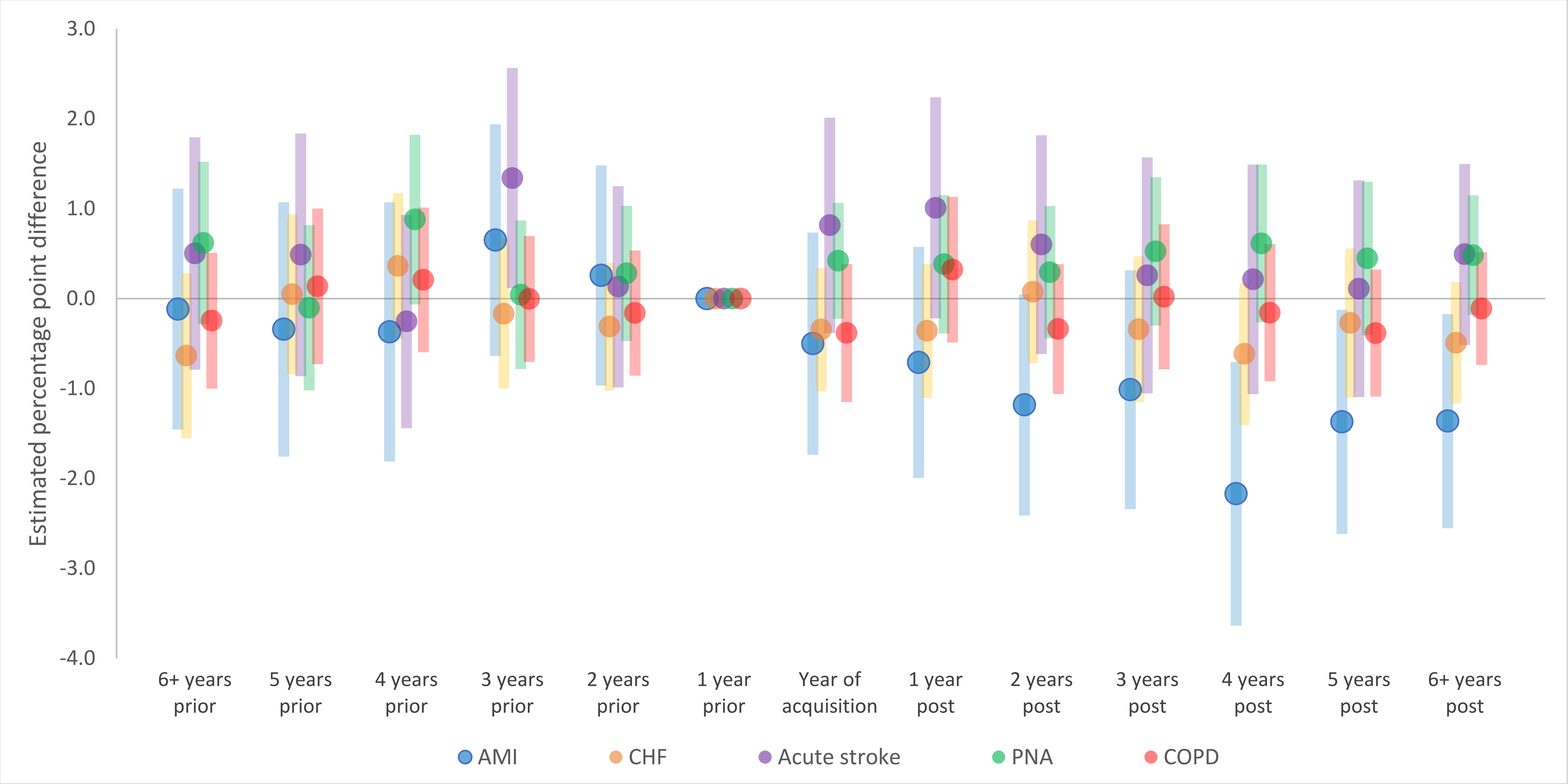

Condition	Pre-acquisition mean	Pre-trend p-value (5-year horizon)	Pre-trend p-value (4-year horizon)
AMI	17.6	0.63	0.49
CHF	11.2	0.18	0.53
Stroke	15.2	0.19	0.12
PNA	12.2	0.27	0.27
COPD	6.8	0.88	0.92

© 2022 Cerullo M et al. *JAMA Network Open*.

eFigure 5. Event Study Plot for Probability of 30-Day Readmission

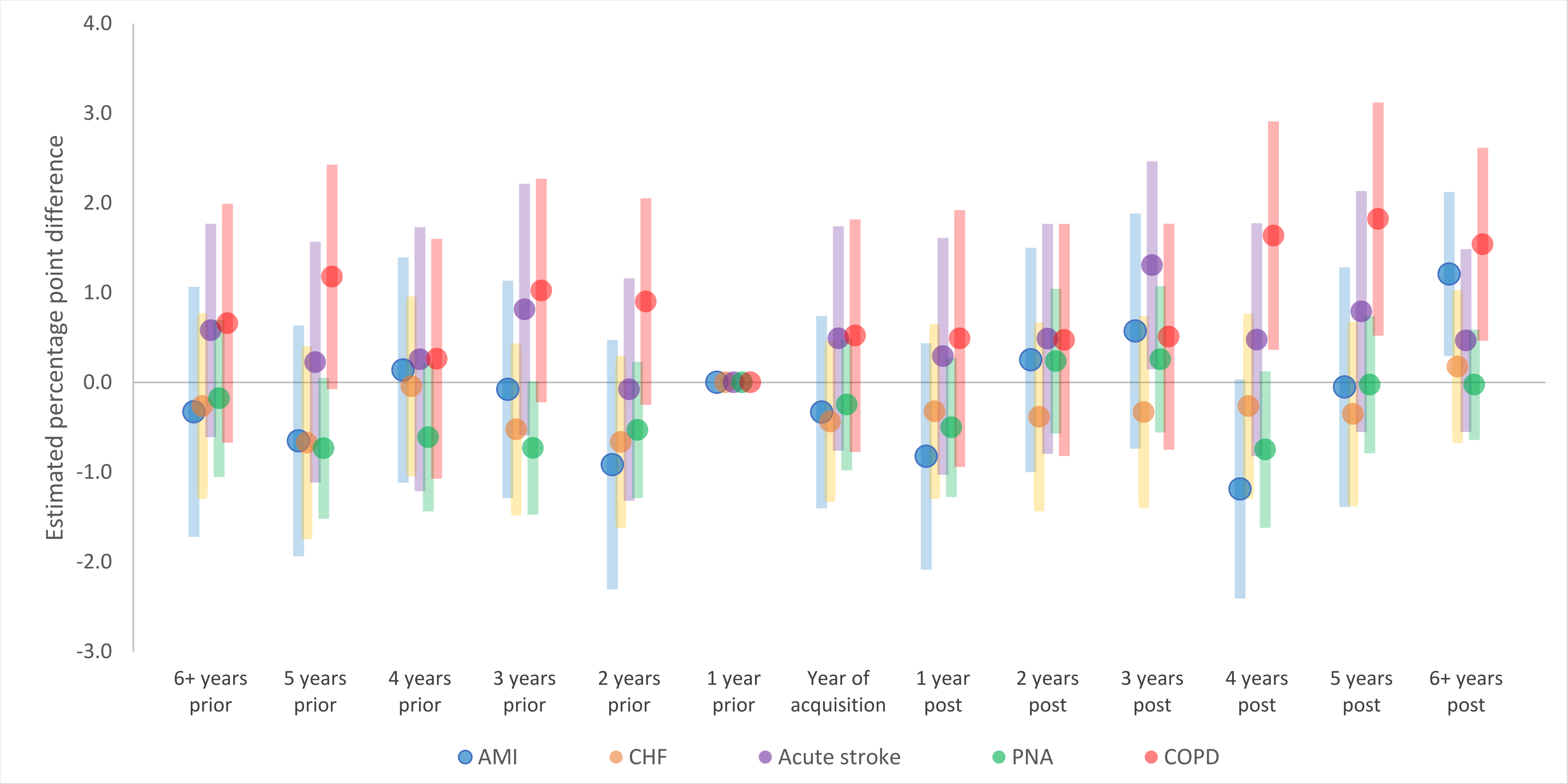

Condition	Pre-acquisition mean	Pre-trend p-value (5-year horizon)	Pre-trend p-value (4-year horizon)
AMI	18.2	0.72	0.61
CHF	19.2	0.48	0.35
Stroke	15.2	0.79	0.75
PNA	12.4	0.28	0.27
COPD	18.0	0.32	0.21

© 2022 Cerullo M et al. *JAMA Network Open*.

eFigure 6. Event Study Plot for Log-Transformed 30-Day Episode Payments

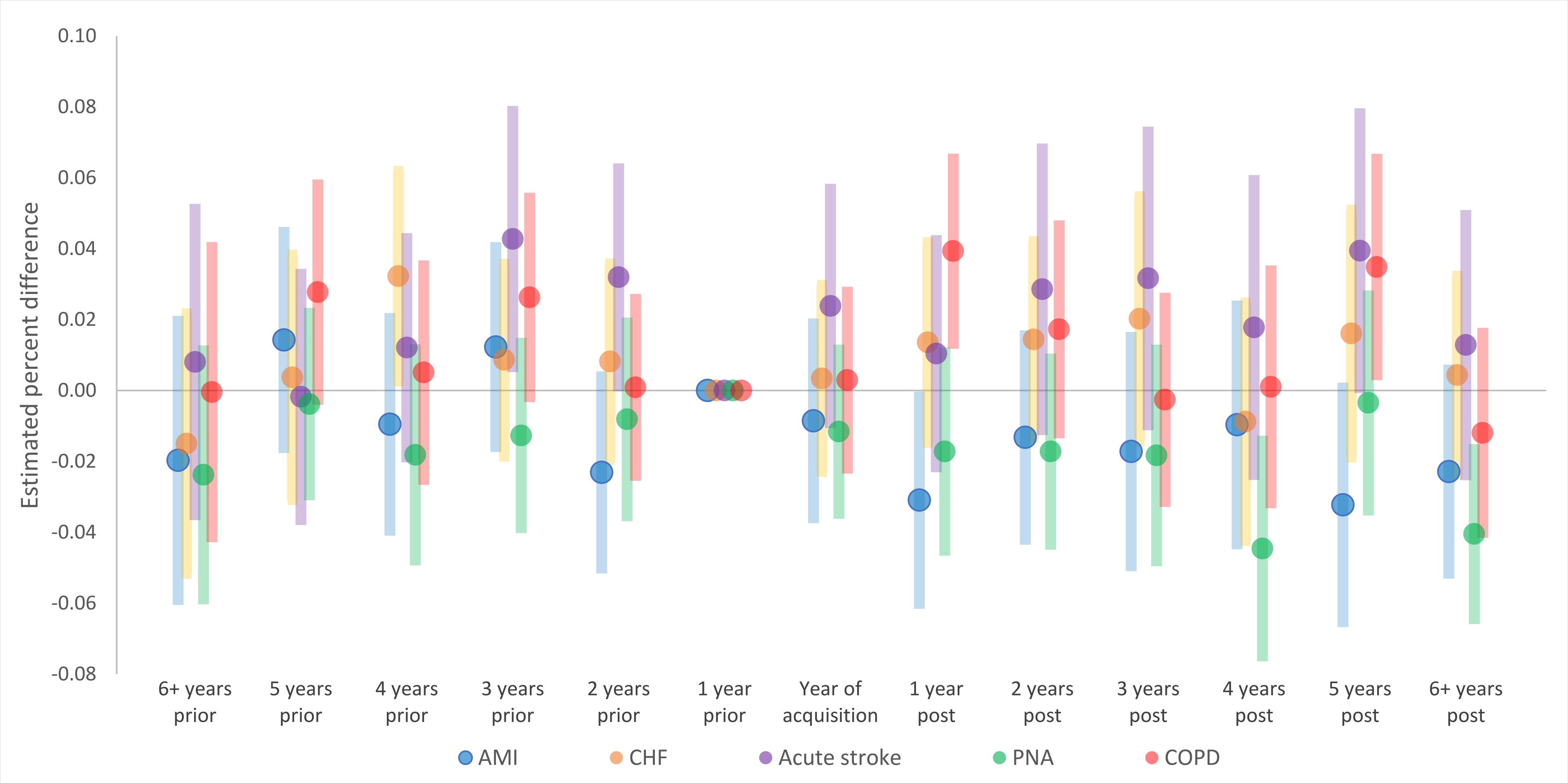

Condition	Pre-acquisition mean	Pre-trend p-value (5-year horizon)	Pre-trend p-value (4-year horizon)
AMI	9.598	0.14	0.13
CHF	9.285	0.06	0.14
Stroke	9.308	0.08	0.05
PNA	9.228	0.79	0.81
COPD	8.913	0.19	0.17

© 2022 Cerullo M et al. *JAMA Network Open*.

eFigure 7. Difference-in-Differences Estimates in Subsample of Hospital Referral Regions With at Least 1 Private Equity Acquisition

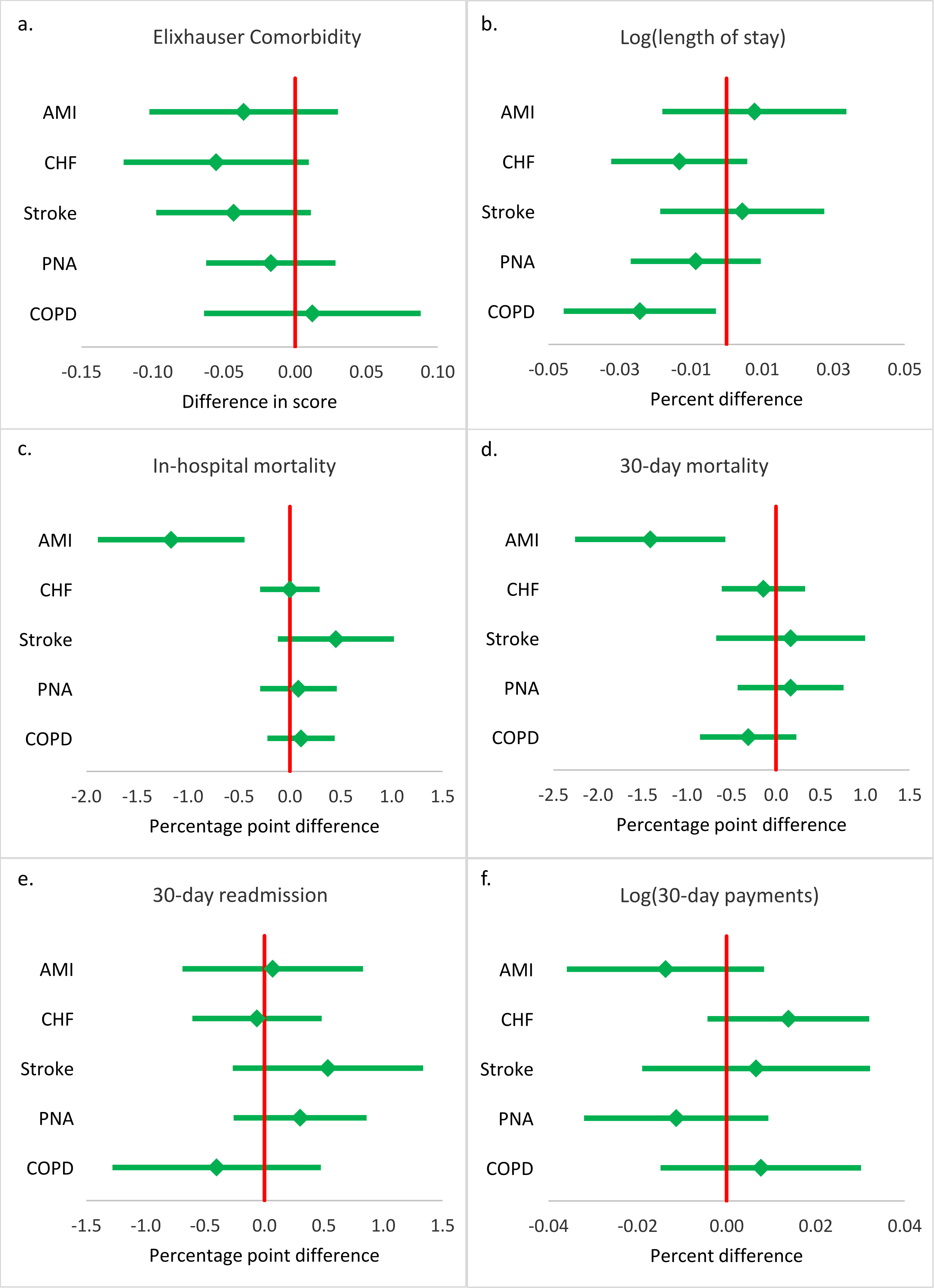

©

eFigure 8. Difference-in-Differences Estimates for HCA and Non-HCA Hospitals

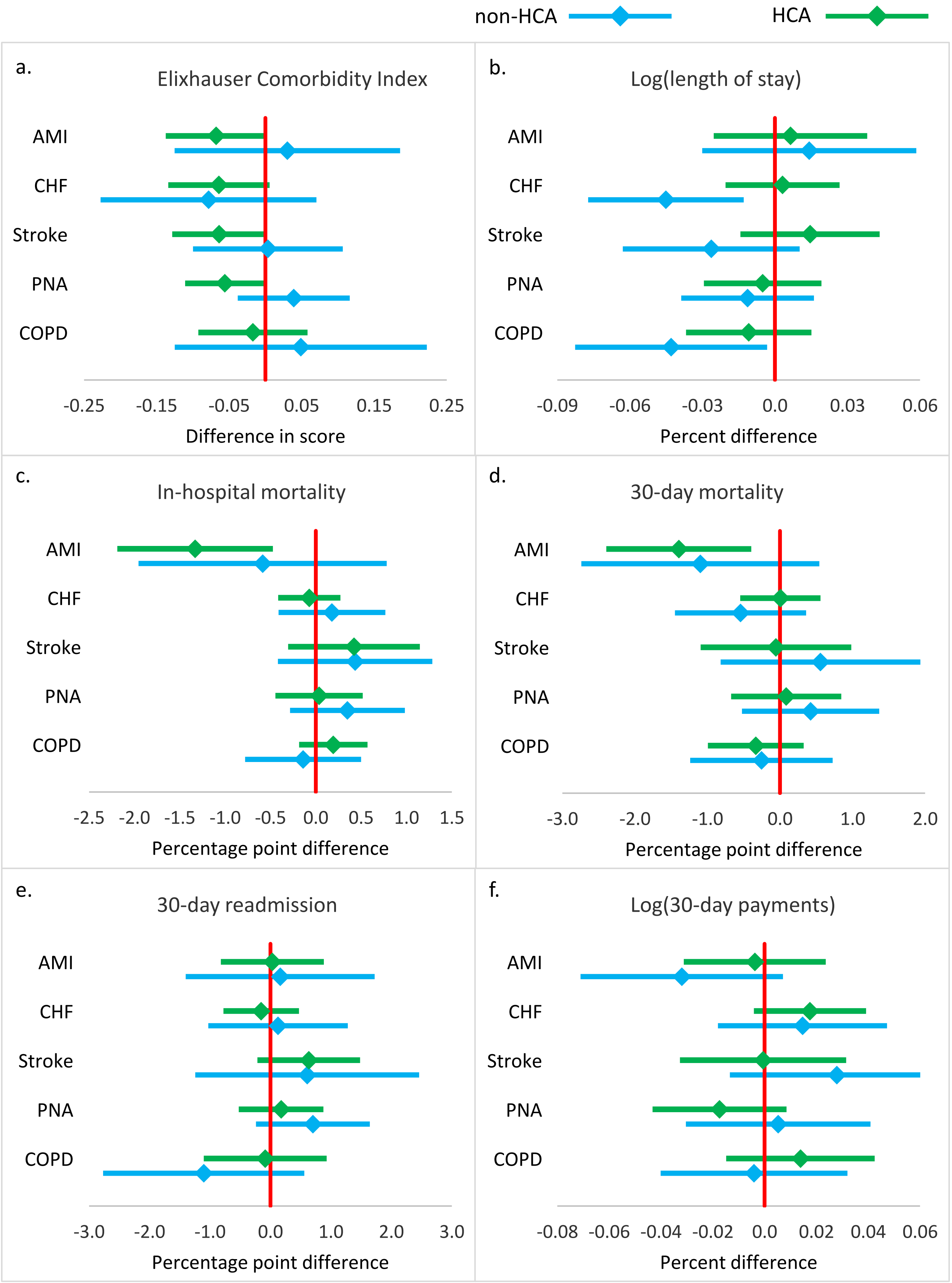

© 2022 Cerullo M et al. *JAMA Network Open*.
